# The Assessment of Immediate Postoperative Delirium in Neurologically Intact Adult Patients Admitted to the Post-anesthesia Care Unit: A Cross-Sectional Study

**DOI:** 10.7759/cureus.29312

**Published:** 2022-09-19

**Authors:** Jyoti Burad, Rohit Date, Mohamed Al Ismaili, Pradeep Sharma, Nigel Kuriakose, Sonali Kodange, Sanath K Birur, Khadija Al Yaqoubi, Ali Al Mawali, Anju Padmalayan, Hatem El Mady, Mohamed Elawdy, Sanjay Jaju, Ali Al Abady

**Affiliations:** 1 Anesthesia and Intensive Care, Sultan Qaboos University Hospital, Muscat, OMN; 2 Urology, Sohar Hospital, Ministry of Health, Sohar, OMN; 3 Medicine, Sultan Qaboos University, Muscat, OMN

**Keywords:** rass, cam-icu, premedication, post-anesthesia discharge, delayed awakening from anesthesia, anesthesia recovery period, postoperative delirium

## Abstract

Background

Immediate postoperative delirium (IPD) in the post-anesthesia care unit (PACU) can cause significant morbidity affecting everyday activities and length of stay with cost implications. This study was undertaken to find the proportion of IPD in PACU and its association with anesthesia and other perioperative factors.

Methods

After obtaining ethical approval and informed consent, this cross-sectional study was conducted in the PACU. A total of 600 consecutive adult patients (American Society of Anesthesiologists (ASA) 1-3) posted for surgery were approached between January and March 2019, of which 402 patients without neurological diseases and language and hearing discrepancies were studied. All patients had the intervention of surgery under anesthesia in a usual manner. Delirium was assessed preoperatively, postoperatively at 15 and 30 minutes, and before discharge from the PACU. IPD was assessed using the Confusion Assessment Method for the Intensive Care Unit (CAM-ICU) score, while sedation/agitation was assessed using the Richmond Agitation-Sedation Scale (RASS). The primary outcomes were the proportion of IPD, association with anesthesia, and perioperative risk factors. The secondary outcomes were the length of stay, delirium treatment, and mortality.

Results

Overall, the IPD proportion was 14.7%. A significant association was demonstrated with premedication with midazolam (odds ration (OR): 3.2; 95% confidence interval (CI): 1.42-7.35; P=0.003), general anesthesia (GA) (OR: 6.3; 95% CI: 2.23-17.8; P<0.001), duration of anesthesia (126 versus 95 minutes; P=0.001), laparoscopic mode of surgical access (OR: 3.4; 95% CI: 1.8-6.4; P<0.001), and postoperative RASS >/< 0 (OR: 10.6; 95% CI: 4.69-24.11; P<0.001) at 30 minutes and before discharge from the PACU. Multivariate analysis showed the strongest association of RASS at 30 minutes with IPD.

Conclusion

The proportion of IPD was found to be 14.7% in this study, and the chances of developing IPD are high if the patient is not awake and calm in the PACU, especially if midazolam is administered as premedication, followed by general anesthesia (GA) for a long duration.

## Introduction

Perioperative events impart a significant strain on the central nervous system (CNS). An imbalance in the central nervous system during the perioperative period can result in an array of consequences including delirium. Many avoidable and unavoidable factors can imbalance CNS perioperatively, such as age, gender, duration and type of surgery, type of premedication, and type of anesthesia. Once delirium sets in, its course might be unaltered despite interventions [1]. This can lead to the loss of everyday activity of normal individuals undergoing even simple surgeries. Thus, the study of causative factors and their prevention is imperative. Delirium is an acute condition characterized by inattention, fluctuating consciousness, and disorganized thinking [2]. Hypoactivity is common in post-anesthesia care unit (PACU) stay, and hence, early postoperative delirium (POD) can be easily missed [3].

POD can lead to higher mortality, increased length of stay, and cognitive dysfunction [4-6]. The association of different types of anesthesia with IPD is so far inconclusive [7]. The effect of perioperative factors can be studied at the earliest in the PACU. Pre-existing confounding factors should be eliminated to highlight the association of perioperative factors with POD. POD developing in the immediate postoperative period during PACU stay has been referred to as immediate postoperative delirium (IPD) in this study. This study aimed to determine the proportion of IPD in the PACU and find the association between the type of anesthesia and other perioperative factors and IPD in neurologically intact adult patients.

## Materials and methods

Study design and setting

This prospective, cross-sectional study was conducted in the PACU of a tertiary care hospital in Oman. Ethical approval of this study was granted by the institutional Medical Research Ethics Committee in December 2018 (MREC #1829). All neurologically intact patients booked for surgery under all types of anesthesia (general, regional, and monitored anesthesia care) between January and March 2019 were enrolled after obtaining written informed consent.

Participant criteria

Adult patients (more than 18 years of age) with American Society of Anesthesiologists (ASA) 1, 2, and 3 status scheduled for elective and emergency surgery under general anesthesia (GA)/regional anesthesia (RA)/monitored anesthesia care (MAC) were included in the study. Exclusion was done for those who refused to consent to the study, with ASA score >/= 4, and with neurological conditions such as dementia, Alzheimer’s disease, psychosis, depression, stroke, head injury, pre-existing delirium, or other known cognitive impairment. Patients with sensory incompetence such as extubation in deep plane of anesthesia leading to unresponsiveness to verbal command and those with auditory and vocal impairment as well as language barrier were excluded from the study.

Conduct of the study

To assess IPD, the Confusion Assessment Method for the Intensive Care Unit (CAM-ICU) score was used as per standard recommendations. The Richmond Agitation-Sedation Scale (RASS) was used to assess sedation and anxiety, and the Numeric Rating Scale (NRS) was used to measure pain. Participant anesthesiologists and nurses underwent training sessions on the proper use of the RASS, NRS, and CAM-ICU scores using the CAM-ICU training manual under the expert guidance of a neurologist. The validated Arabic version of the CAM-ICU score was used [8]. The details of the study were explained to each patient with the help of an information sheet by a dedicated team of nurses and doctors who were not involved in administering anesthesia to these patients. Written informed consent was obtained, and preoperative scores of pain, anxiety, agitation, and delirium were measured. Anesthesiologists, who were not associated with the current research, performed a baseline pre-anesthesia checkup. Anesthesia intraoperative and postoperative management was performed according to the standard institutional protocol, and details were recorded. Once the patient was transferred to the PACU, RASS, CAM-ICU, and NRS scores were evaluated 15 and 30 minutes after the end of anesthesia exposure and prior to discharge from the PACU by trained nurses. IPD onset was determined in a single postoperative episode in the PACU. If the patient had RASS ≥-3, then the CAM-ICU score was used to diagnose IPD. Patients with a CAM-ICU score ≥ 3 at any of the three points of time (15 minutes, 30 minutes, and discharge) in the PACU were diagnosed with IPD.

Outcomes

The primary outcomes of this study were the proportion of IPD in the PACU and the association with the type of anesthesia and other perioperative factors. The secondary outcomes included treatment for IPD, complications, postoperative length of hospital stay, and mortality.

Potential predictors

Patient-specific data such as age and gender, along with information including comorbidities such as diabetes, hypertension, acute kidney injury, chronic kidney dysfunction, ischemic heart disease, bronchial asthma, dyslipidemia, and sickle cell disease, and ongoing preoperative medication details, were recorded. Details of anesthetic and surgical management such as the type of anesthesia, premedication, anesthetic medications, surgical access (endoscopic, laparoscopic, or open), urgency of surgery, and type of surgery were documented for all patients. The proforma was handed over to the principal investigator. Patient identity was coded, and data were statistically analyzed.

Bias

Bias was avoided by training a separate team to assess IPD under the guidance of an experienced neurologist and exclusion of patients with pre-existing neurological conditions as well as pre-existing delirium.

Sample size

A pilot study of 24 cases showed the proportion of IPD to be approximately 25%. Based on a population size of 600 eligible patients during the study period, a level of confidence of 95%, and relative precision of 10% on either side, the optimum sample size was calculated as 356 patients. We decided to enroll nearly 10% more subjects to compensate for the inadvertent data loss resulting in a minimum requirement of 395 subjects. In total, 402 consecutive patients were finally studied.

Statistical methods

The collected data were coded, tabulated, and analyzed using Statistical Package for the Social Sciences (SPSS) version 23.0 (IBM Corp., Armonk, NY, USA) and MedCalc Statistical Software version 19.6 (MedCalc Software bv, Ostend, Belgium). Sociodemographic variables and other risk factors were categorized, and their frequencies and percentages were stated across age groups. The significance of association was tested using the χ^2^ test for all categorized variables. Their odds ratios (ORs) and confidence intervals (CIs) were deduced wherever applicable. For abnormally distributed data such as age, duration of anesthesia, and postoperative length of stay, medians and interquartile ranges were calculated for IPD and no IPD groups and were analyzed using the Mann-Whitney U test. A P value < 0.05 was considered statistically significant.

To build the regression model, potentially explanatory variables were identified at the protocol stage that may be significantly related to the response variable. To determine whether the variables may be included in the regression analysis, exploratory data analysis was done as the first step. A simple univariate analysis was done using the χ^2^ test to compare IPD with the categorical variables, and analysis of variance (ANOVA) was used to compare the IPD to the continuous variables. All potential explanatory variables were tested for collinearity using correlation analysis to determine whether the values of any two of the variables were associated. An accompanying correlogram was drawn. Variables with only minimal or moderate correlation were treated as independent of each other and included in the analysis. There was a moderate correlation between 30-minute RASS and GA/no GA; the other variables had minimal correlation, so these variables were continued in the analysis. There was no obvious interaction noted among any of the finally included variables. They were treated as independent of each other.

## Results

Patient characteristics

**Figure 1:**
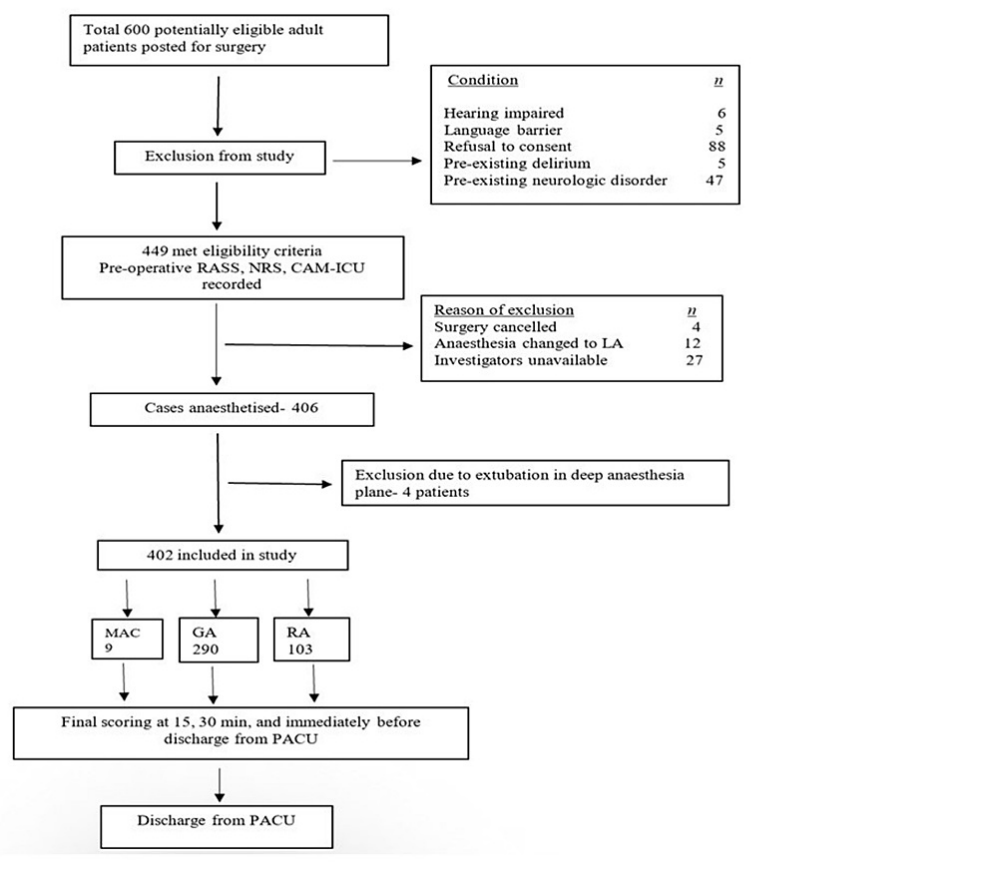
Flow of Patients During the Study RASS: Richmond Agitation-Sedation Scale; NRS: Numeric Rating Scale; CAM-ICU: Confusion Assessment Method for the Intensive Care Unit; LA: laparoscopic approach; MAC: monitored anesthesia care; GA: general anesthesia; RA: regional anesthesia; PACU: post-anesthesia care unit

Altogether, 600 adult patients were posted for surgery, of which 449 patients fulfilled the study criteria and consented to participate. Eventually, 402 patients could be included in the study (Figure 1). Relevant patient characteristics are highlighted in Table 1. In total, 290 patients received GA, 103 RA, and nine MAC. To segregate the effect of pre-existing and established risk factors, cumulative preoperative factors’ risk stratification was deduced. The presence of any two of the established factors such as emergency surgery [9], ASA grade >/= 2 [10,11], and 4-6 preoperative medications [12] were considered high risk for the development of IPD. There were 55 patients identified to be at high risk for developing IPD.

**Table 1:** Patient Characteristics GA: general anesthesia; RA: regional anesthesia; MAC: monitored anesthesia care; SD: standard deviation; ENT: ear, nose, and throat specialty

Demographic parameter		Total (number (%))	18-35 years (number (%))	36-60 years (number (%))	>60 years (number (%))	P value (χ^2^)
Age (years)	Mean (SD)	42.38 (15.48)	28.53 (5)	46 (7)	69.65 (6)	-
Gender	Male	146 (36)	55 (33)	55 (32)	36 (57)	0.001
	Female	256 (64)	112 (67)	117 (68)	27 (42.9)	
Type of surgery	ENT, ophthalmology, dental	39 (10)	23 (14)	10 (6)	6 (9)	<0.001
	Gynecology	59 (15)	31 (19)	27 (16)	1 (2)	
	Orthopedics	22 (5)	9 (5)	3 (2)	10 (16)	
	General surgery	90 (22)	34 (20)	48 (28)	8 (13)	
	Urology	14 (3)	2 (1)	4 (2)	8 (13)	
	Minor	178 (44)	68 (41)	80 (46)	30 (47)	
Urgency of surgery	Emergency	32 (8)	21 (13)	7 (4)	4 (6)	0.013
	Elective	370 (92)	146 (87)	165 (96)	59 (94)	
Surgical approach	Open	269 (67)	106 (63)	121 (70)	42 (67)	0.282
	Laparoscopy	61 (15)	31 (19)	24 (14)	6 (10)	
	Endoscopy	72 (18)	30 (18)	27 (16)	15 (23)	
ASA grade	ASA 1	171 (43)	97 (58)	68 (39)	6 (10)	<0.001
	ASA 2	198 (49)	69 (41)	96 (56)	33 (52)	
	ASA 3	33 (8)	1 (0.6)	8 (5)	24 (38)	
Risk of delirium	Low risk	347 (86)	160 (96)	153 (89)	34 (54)	<0.001
	High risk	55 (14)	7 (4)	19 (11)	29 (46)	
Number of preoperative medications	<3	363 (90)	160 (96)	158 (92)	45 (71)	<0.001
	4-6	34 (9)	7 (4)	12 (7)	15 (24)	
	>6	5 (1)	0 (0)	2 (1)	3 (5)	
Type of anesthesia	GA	290 (72)	127 (76)	137 (80)	26 (41)	<0.001
	RA	103 (26)	36 (22)	32 (19)	35 (56)	
	MAC	9 (2)	4 (2)	3 (2)	2 (3)	

Primary outcomes

**Figure 2:**
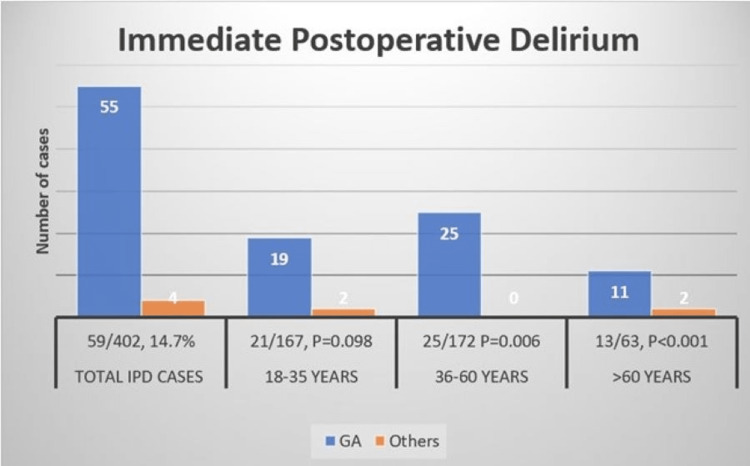
Incidence of Immediate Postoperative Delirium IPD: immediate postoperative delirium; GA: general anesthesia

There was no significant effect of age on IPD with the median age being 45 years (SD: 16.6 years) in IPD patients and 42 years (SD: 15.2 years) in non-IPD patients. The presence of cumulative preoperative factors’ risk did not make a significant impact on the proportion of IPD. The proportion of patients with IPD was found to be 14.7% (59/402) in the PACU. The incidence of IPD was found to be highest in the older age group (P<0.001), lower in middle age (P=0.006), and lowest in young ones (P=0.098) (Figure 2).

Effect of Anesthesia

Patients who received midazolam had a higher proportion of IPD (52/291, 18%) (P=0.003). Out of these 52 patients, 49 patients received GA with midazolam. The proportion of IPD was relatively higher with GA (55/290, 19%) (OR: 6.3; 95% CI: 2.2-17.8; P=0.00) as compared to the other types of anesthesia. The duration of anesthesia was higher for the IPD group versus the non-IPD group (median: 126 (86-180) versus 95 (63-134) minutes; P=0.001).

Effect of Surgery

The proportion of IPD was highest for patients undergoing general surgery (23/90, 26%). All these cases received GA. Of the patients who underwent orthopedic procedures, 6/22 (27%) cases received GA only, out of which, 3/22 (14%) developed IPD. When the surgical access was laparoscopic, 19/61 (31%) developed IPD as compared to other accesses for surgery (OR: 3.4; 95% CI: 1.8-6.4; P=0.00).

Other Factors

Other perioperative confounding factors including the presence of a urinary catheter, nasal pack, or hypotension were not significantly associated with IPD.

The association of perioperative factors with IPD is highlighted in Table 2.

**Table 2:** Association of Different Factors With IPD IPD: immediate postoperative delirium; IPD+: immediate postoperative delirium positive; IPD-: immediate postoperative delirium negative; Sig: significance; DOA: duration of anesthesia; OR: odds ratio; CI: confidence interval; IQR: interquartile range; ENT: ear, nose, and throat specialty; MAC: monitored anesthesia care *Mann-Whitney U test

Factors		IPD+ (number (%))	IPD- (number (%))	OR (CI)	P value
Age	Median (IQR)	42 (32-56)	38 (31-52)	-	0.205^*^
Gender	Male	24 (16)	122 (84)	1.24 (0.70-2.1)	0.451
	Female	35 (14)	221 (86)		
Type of surgery	ENT, dental, ophthalmology	6 (15)	33 (85)	-	0.027
	Gynecology	4 (7)	55 (93)		
	Orthopedics	3 (14)	19 (86)		
	General surgery	23 (26)	67 (74)		
	Urology	2 (14)	12 (86)		
	Minor surgery	21 (12)	157 (88)		
Surgical access	Laparoscopic	19 (31)	42 (69)	3.40 (1.80-6.42)	0.000
	Non-laparoscopic	40 (12)	301 (88)		
Urgency of surgery	Emergency	2 (6)	30 (94)	0.366 (0.08-1.57)	0.160
	Elective	57 (15)	313 (85)		
ASA grade	1	24 (14)	147 (86)	-	0.952
	2	30 (15)	168 (85)		
	3	5 (15)	28 (85)		
Total preoperative medications	<3	50 (14)	313 (86)	-	0.290
	4-6	8 (24)	26 (76)		
	>6	1 (20)	4 (80)		
Pre-existing risk of IPD	Minimum	47 (13)	300 (87)	0.561 (0.27-1.14)	0.107
	High	12 (22)	43 (78)		
Premedication	Midazolam	52 (18)	239 (82)	3.23 (1.42-7.35)	0.003
	No midazolam	7 (6)	104 (94)		
Type of anesthesia	General	55 (19)	235 (81)	6.31 (2.23-17.88)	0.000
	Others (regional + MAC)	4 (4)	108 (96)		
Duration of anesthesia	Minutes (median (IQR))	126 (86-180)	95 (63-134)	-	0.001^*^

Pain and Sedation Levels

The association of IPD with NRS and RASS is shown in Table 3. NRS did not affect IPD, whereas whenever RASS was </> 0, it was associated with an increased proportion of IPD.

**Table 3:** Association of IPD With Preoperative and Postoperative Pain and Sedation Scores IPD: immediate postoperative delirium; IPD+: immediate postoperative delirium positive; IPD-: immediate postoperative delirium negative; Sig: significance; NRS: Numeric Rating Scale; RASS: Richmond Agitation-Sedation Scale; PACU: post-anesthesia care unit

Timeline of measurement	Score	Value	IPD+ (number (%))	IPD- (number (%))	P value
Preoperative	NRS	0-3	58 (15)	334 (85)	0.771
		4-6	1 (14)	6 (86)	
		7-10	0 (0)	3 (100)	
	RASS	<0	0 (0)	3 (100)	0.445
		0	50 (14)	304 (86)	
		>0	9 (20)	36 (80)	
15 minutes post-anesthesia	NRS	0-3	50 (14)	298 (86)	0.429
		4-6	7 (15)	41 (85)	
		7-10	2 (33)	4 (67)	
	RASS	<0	41 (20)	164 (80)	0.000
		0	2 (1)	149 (99)	
		>0	16 (35)	30 (65)	
30 minutes post-anesthesia	NRS	0-3	56 (15)	324 (85)	0.916
		4-6	3 (14)	18 (86)	
		7-10	0 (0)	1 (100)	
	RASS	<0	45 (26)	130 (74)	0.000
		0	7 (3)	202 (97)	
		>0	7 (39)	11 (61)	
Before discharge from the PACU	NRS	0-3	59 (15)	338 (85)	0.647
		4-6	0 (0)	3 (100)	
		7-10	0 (0)	2 (100)	
	RASS	<0	35 (32)	73 (68)	0.000
		0	15 (5)	263 (95)	
		>0	9 (56)	7 (44)	

Secondary outcomes

Postoperative length of stay did not differ significantly between the IPD group and the no-IPD group (2 days versus 1 day; P=0.156). Postoperative complications were observed in 11/402 (2.7%) cases. More than half of these cases had IPD (6/11, 54%). Treatment for IPD was required for 10/59 (17%) patients. One patient developed POD after discharge from PACU, for which treatment was given. Mortality was observed in one patient who underwent multiple surgeries and consequently had a long hospital stay and septic shock.

Multivariate logistic regression analysis

For multivariate analysis, based on preliminary analysis, it was deduced that the following variables may influence IPD: age, duration of anesthesia, GA technique, premedication with midazolam, laparoscopic surgical access, and RASS at 30 minutes. These six potential variables were entered into the model without checking. Under the principle of parsimonious data modeling, further unnecessary variables were discarded. Using a backward selection method, five of the six variables were fitted serially (sequentially removing one variable each time), observing obvious trends. Variables that were observed to be consistently eliminated from the resultant regression analysis were culled. A three-variable model was finally arrived at, predicting 85% of the results. All the potential variables were tested for collinearity by correlation analysis to determine the association of any two variables. An accompanying correlogram was drawn. There was a moderate correlation between 30-minute RASS and GA/no GA and minimal correlation between the other variables; thus, these variables were analyzed. As there was no obvious interaction noted among any of the finally included variables, they were treated as independent of each other. The highest causative association was observed between IPD and RASS </> 0 at 30 minutes (OR: 9.46; 95% CI: 4.11-21.75; P<0.00).

## Discussion

Proportion of IPD

The proportion of IPD in this study was lower than in others [13]. A study on POD after hip fracture repair reported a prevalence of recovery room delirium of 45%, but the mean age of their subjects was 77 years, and the type of surgery was limited to hip fracture repair under GA unlike the present study [12]. Premedication and the effects of anesthesia in addition to other well-known factors can lead to a higher proportion of IPD [14].

Perioperative factors

The focus of the present study design was to find an association between perioperative factors and IPD. Pre-existing neurological impairment and cognitive dysfunction can lead to prolonged cognitive disability [15] and higher POD, and therefore, these patients were excluded to highlight the novel neurological impairments. This could explain our study results. Surprisingly, there was no association found between the established risk factors such as comorbidities, emergency surgery, and preoperative medications with IPD in this study [16,17]. This might be explained by the fact that the sample size was not calculated to study the effect of these factors on the IPD.

Surgical factors

It has been reported earlier that elderly patients undergoing orthopedic procedures have a higher proportion of POD, but the results of the present study contradict this finding [7,18,19]. In this study, nine out of 10 patients who underwent orthopedic surgery received RA, which explains this difference. A recent study comparing RA and GA for elderly patients with hip fractures demonstrated identical results [20]. A previous study on elderly patients undergoing gastrectomy showed that the laparoscopic approach did not offer a reduced proportion of POD when compared to the open surgical approach [21]. The present study however finds the laparoscopic mode of surgical access rather resulted in increased IPD across all age groups, especially in the elderly. This could be explained by the higher risk of IPD with hypercapnia and residual pneumoperitoneum [22,23]. Focused studies on the effect of the laparoscopic mode of surgical access on IPD are warranted to better understand the causative association.

Anesthetic factors

Patients who received anti-anxiety premedication (midazolam) developed a significantly higher proportion of IPD. Those who received midazolam and GA developed more IPD. This can be due to the synergistic effect of the drug with GA, potentiating the sedative effect.

It has been previously speculated that RA has an advantage over GA [24], but the evidence was largely inconclusive [25]. Recently, a study [20] highlighted the advantages of RA over GA in terms of POD. The present study reaffirms the advantage of RA over GA.

The duration of anesthesia was significantly higher for IPD cases in the present study. Similar results were shown in a recent study by Ravi et al. [26]. They reported that for every 30-minute increment in the duration of surgery for hip fixation, the delirium risk increased by 6%, and this risk was higher with GA [26].

Pain and sedation levels

The CAM-ICU score has been validated to diagnose IPD [27]. Also, it is documented to be better than the Intensive Care Delirium Screening Checklist (ICDSC); hence, CAM-ICU was used for the present study [28]. Inexplicably, the NRS scores were not particularly different between the patients with and without IPD. Identical observations were made in a study on gastrectomy patients by Shin et al. [21]. Contrarily, the sedation/agitation level demonstrated a significant association with IPD when postoperative RASS deviated from a score of 0. A significant association of abnormal RASS (</=-2 and >/=1) with POD has been demonstrated previously [29]. A longer duration of anesthesia has been found to be associated with abnormal RASS and POD [30].

Interpretation

This study highlights that the proportion of IPD was 14.7%, and it can be limited if patients are awake, calm, and comfortable in the immediate postoperative period during PACU stay. Avoidance of IPD might also be achieved by opting for regional anesthesia preferentially, limiting the duration of anesthesia, and judicious use of antianxiety premedication and laparoscopic mode of surgical access, but further focused studies are needed to confirm these speculations.

Generalizability

The results of this study can be reproduced, as the patient population was largely prototypical, anesthetic management was standard, and the staff was trained for the proper obtainment of scores.

Strength of the study

The present study purely highlights the effect of perioperative factors on IPD in a wide variety of everyday surgeries, excluding the confounding effects of pre-existing neurological ailments.

Limitation

The exclusion of patients with pre-existing neurological ailments might have affected the proportion of IPD. However, as the aim of the study was to find out the impact of perioperative events on IPD, this exclusion was essential. This was a single-centered observational study, and hence, there might have been some inherent institutional practices or environmental factors that might have affected the results.

## Conclusions

This study shows that the proportion of IPD was 14.7% and that the chances of developing IPD are high if patients are not awake and calm in the PACU with a deviation of postoperative 30-minute RASS from 0. Monitoring of delirium in the early postoperative period in the PACU should be a routine practice. Balancing the use of anesthesia such that patients are calm and awake in the PACU can prevent the development of immediate postoperative delirium. The judicious use of premedication with midazolam, GA, and laparoscopic mode of surgical access is advocated. Limiting the duration of surgery and anesthesia as well as opting for regional anesthesia (RA) and monitored anesthesia care (MAC) whenever possible can also be beneficial.
